# Practical Points

**Published:** 1912-02-03

**Authors:** 


### PRACTICAL POINTS.

(Criticism and Suggestions Invited.)

### II.—Short Circuits in Electric Cables.

The insulating envelope of a wire or cable is the weakest part of the whole system; and if it is exposed to mechanical injury it always suffers, shorts or other troubles resulting. The common source of shorts from mechanical injury is the carelessness or ignorance of workmen in other trades. Wires or cables have been laid in the plaster and carefully covered up; and a plumber, a bell-fitter, or some other workman has been called in to do some repairs or alterations. He may not know of the existence of the wires or cables. He may not have had any experience of work in buildings where electric light or power wires or cables are fixed; or he may be simply careless. In either case he sometimes drives his chisel through the steel tube or wood casing that protects the wires, and through the insulating envelope of the cable or wire, usually connecting the two together in the process. He may drive a nail in the same way; he may merely cut the cable off and leave the ends exposed, and they get knocked into contact either by the workman who has cut them off or someone who happens to be passing and is carrying some object which knocks against them. The result is the same; a short is formed, with the troubles already mentioned. In the case of lead-covered paper cables, the ignorant or careless workman doing repairs may also produce the trouble that the lead tube is designed to protect the paper against. As explained in a previous article, paper is an insulator as long as it is dry. After impregnation with resinous oils, it remains an insulator so long as moisture does not enter. Nothing happens so long as the outer protecting lead tube is intact. If that be punctured, however, the moisture in the atmosphere enters at the puncture, and works its way along the capillary space between the paper and the lead tube until it reaches the weak point. There it gradually causes deterioration of the insulation till, when one of those sudden heavy rises of pressure mentioned in the previous article above occurs, a spark passes across, the insulation is broken down, the lead covering is fused to the copper conductor, and if the lead covering is in contact with the iron or steel containing-tube, a short may result.

Sometimes the staples that are used for fixing electric-bell wires are employed to hold up a flexible cord employed to deliver current to a lamp or small motor. No harm may be done at first, if the staple is loose; but the cord chafes on the staple, gradually rubs through its covering, and a short is formed between the two flexible conductors in the cord, sometimes by way of the staple and sometimes directly. This is perhaps one of the most serious cases of short, because the cover of the flexible cord is made of very inflammable material, the conductor itself is comparatively small, and a short leads to the liberation of a quantity of heat.

A variation of this, again, may be brought about by the practice, which is somewhat common, of tying knots in the flexible cord to shorten it. The covering of the cord is chafed through in course of time at some part of the knot by the vibration which is present in all buildings, and the conductors of the flexible cord being particularly liable to be severed, some of them are parted, their sharp ends push their way through the covering, make connection with other sharp ends that have pushed their way through the other cord, and a short is the result.

Another variation is the imperfect construction of the arrangements of pendant lamps that can be raised or lowered, on similar lines to the old gas chandelier. The cord is made long enough to allow the lamp to come down sufficiently near the desk or table to throw a good light over a certain limited portion, as for writing, for operating, or for delicate work. When not in use the lamp is pushed up out of the way, the surplus length being taken up by passing over pulleys, a balance-weight being attached to a pulley at the loop.

If the pulleys contain any sharp edges, if, as sometimes happens, minute burrs have been left on their surfaces, even if the groove of the pulley is not sufficiently large, if the circle of the pulley has not a sufficiently large radius, and if either the lamp is pushed up and down frequently or remains for a long time in one position so that the cord is bent rather sharply at one point, the covering of the cord may be weakened, portions of the flexible conductor may be severed and may push their way through the cord, meeting other pieces of the other conductor, a short being the result.

Buildings, it will be remembered, are not always constructed on the best possible lines. Architects sometimes make mistakes in their calculations of the stresses and strains which beams, girders, etc., will stand. Again, the old tale of the lowest price and the cheap constructor comes in, and it may happen that a beam has been fixed which is not of sufficient strength to maintain its horizontal position, or in the case of a vertical member to maintain its vertical position. In such cases the pipe or wood casing which is fixed under such a beam, or against such a vertical member, may be exposed to strains which may either force the metal of the conduit through the insulating covering of the wire it is protecting, or force the lead covering of the paper cable through its paper, or, again, may burst open the wood casing and strain the conducting wires in a similar manner. There are a number of cases of this kind where two conductors may be brought directly into connection, or may be brought one into connection with a metal at one point, and another into connection with a metal metallically continuous with the first at another point, and a short occurs.

